# Whole-Genome Sequencing and Bioinformatics Analysis of *Apiotrichum mycotoxinivorans*: Predicting Putative Zearalenone-Degradation Enzymes

**DOI:** 10.3389/fmicb.2020.01866

**Published:** 2020-08-03

**Authors:** Jinyuan Sun, Yan Xia, Dengming Ming

**Affiliations:** College of Biotechnology and Pharmaceutical Engineering, Nanjing Tech University, Nanjing, China

**Keywords:** *Apiotrichum mycotoxinivorans*, whole-genome sequencing, mycotoxin detoxification, zearalenone (ZEA), BVMO, carboxylesterase, genome-scaled prediction of substrate-specific enzyme (GPSE)

## Abstract

Biological detoxification techniques have been developed by using microorganisms such as bacteria, yeast, and fungi to eliminate mycotoxin contamination. However, due to the lack of molecular details of related enzymes, the underlying mechanism of detoxification of many mycotoxins remain unclear. On the other hand, the next generation sequencing technology provides a large number of genomic data of microorganisms that can degrade mycotoxins, which makes it possible to use bioinformatics technology to study the molecular details of relevant enzymes. In this paper, we report the whole-genome sequencing of *Apiotrichum mycotoxinivorans* (*Trichosporon mycotoxinivorans* in old taxonomy) and the putative Baeyer-Villiger monooxygenases (BVMOs) and carboxylester hydrolases for zearalenone (ZEA) degradation through bioinformatic analysis. In particular, we developed a working pipeline for genome-scaled prediction of substrate-specific enzyme (GPSE, available at https://github.com/JinyuanSun/GPSE), which ultimately builds homologous structural and molecular docking models to demonstrate how the relevant degrading enzymes work. We expect that the enzyme-prediction woroflow process GPSE developed in this study might help accelerate the discovery of new detoxification enzymes.

## Introduction

Many secondary fungal metabolites produced by a variety of molds from *Fusarium*, *Aspergillus*, and *Penicillium* are highly toxic and make up the majority of mycotoxins (Capcarova et al., 2016). In addition to the direct consequences of huge economic losses, the ubiquitous presence of mycotoxins in food and feed products also increases the risk of serious diseases, such as cancer, anamorphosis, dermatopathya, nephroma, and other non-specific symptoms, in both animal and human (Peraica et al., 1999; Hussein and Brasel, 2001). Therefore, a large number of tools are needed to remove a variety of mycotoxins from food and feed. Over the years, a wide spectrum of decontamination strategies have been developed by using physical [thermolysis, radiation treatment, etc. (Karlovsky et al., 2016)], chemical [oxidation, reduction, hydrolysis, absorption, etc. (Danicke et al., 2012)] and biochemical [by using biological agents (Vanhoutte et al., 2016)] techniques. Among these methods, the biodegradation of mycotoxins has become the most concerned method because of its cost-efficiency and green environmentally environmental friendliness. A number of microorganisms including bacteria, fungi, and yeasts have been found to be biodegradable, and the underpinning mechanisms are still under study.

The workhorses for microbial degradation are enzymes that efficiently convert mycotoxins into non-toxic or less toxic compounds (Ben Taheur et al., 2019). Therefore, it has become an indispensable task in the field of biodegradation to search for detoxifying enzymes and to characterize their catalytic activities, physicochemical properties, and three-dimensional structures (Loi et al., 2017; Lyagin and Efremenko, 2019). For example, aflatoxins are among the most toxic mycotoxins produced by fungus *Aspergillus flavus* and *Aspergillus parasiticus*, causing food and feed pollution and resulting in human cancers (Kuiper-Goodman, 1995). After decades of in-depth worldwide research, a bunch of enzymes from various organisms, such as bacteria, fungi, mammals, have been identified with aflatoxin biodegradability (Ben Taheur et al., 2019; Lyagin and Efremenko, 2019). Most of these enzymes are classified as aflatoxin dialdehyde reductase, which is characterized by the introduction of hydroxyl groups at C8 and C9 to open the bifuran ring (Kozma et al., 2003). This biotransformation is essential in eliminating aflatoxin’s tumorigenicity. Enzymes that cut the aflatoxin coumarin ring was also identified, although the involved mechanisms are still unknown because of the lack of protein complex structures (Marimón Sibaja et al., 2019). In recent years, computer modeling technology has become more and more popular in the search for the next generation of aflatoxin degrading enzymes (Dhakal et al., 2017; Zarev et al., 2020).

Another important example is zearalenone (ZEA), a mycotoxin mainly produced by *Fusarium*, which happens to infect maize, barley, wheat, sorghum in various cool and humid seasons. The hormone-like structure of ZEA leads to hyperestrogenism symptoms, resulting in infertility and economic loss of animal husbandry. A carboxylesterase gene (ZHD101) has been reported to deactivate ZEA by opening the lactone ring (el-Sharkawy and Abul-Hajj, 1988; Takahashi-Ando et al., 2002), which was later verified by the X-ray crystal structures of enzyme-substrate and enzyme-product complexes (Peng et al., 2014; Qi et al., 2017). This enzyme is critically important for *Clonostachys rosea* to protect the plant from the wild type ZEA-producer *Fusarium graminearum* (Kosawang et al., 2014). However, the degradation products α– and β–zearalenol (ZOL) still remain estrogenic biotoxicity (Vekiru et al., 2010). Peroxiredoxin and homologs were reported to non-specifically act on ZEA by oxidizing the double bond at C1’/C2’ and/or hydroxy-groups at C2 and C4 (Yu et al., 2012; Tang et al., 2013; Garcia et al., 2018). Vekiru et al. (2010) characterized the structural and estrogenic activity of the ZEA degradation products processed by *Trichosporon mycotoxinivorans* (*T. mycotoxinivorans*), a basidiomycete yeast used as a microbial feed additive against mycotoxins (Molnar et al., 2004; Schatzmayr et al., 2006). In their study, the main products of ZEA were identified and characterized by liquid chromatography-tandem mass spectrometry (LC- MS/MS) and LC-diode array detector (DAD) analysis. Their analysis suggested that ZEA was first oxidized by a Baeyer-Villiger oxidase (BVMO), resulting in the insertion of an oxygen atom between C5’ and C6’. The ring structure of this intermediate metabolite was then breaking at the newly formed ester bond with a carboxylesterase. However, both the BVMO and carboxylesterase enzymes in this degradation pathway remained unclear, which hindered their application in ZEA degradation.

In this paper, we report the whole-genome sequencing and annotation of *T. mycotoxinivorans* (Molnar et al., 2004), a strain recently renamed *Apiotrichum mycotoxinivorans* by Liu and collaborators in an integrated phylogenetic classification of the Tremellomycetes in 2015 (Liu et al., 2015). We then screened candidate BVMO and craboxylesterase genes for the ZEA-degradation with the GPSE workflow process. Homologous modeling and molecular docking are used to investigate the potent substrate-enzyme interactions. Pathogenomics of *A. mycotoxinivorans* were also investigated based on genome annotation, since the emergence of invasive infections have been recently reported (Almeida et al., 2017; Dabas et al., 2017).

## Materials and Methods

### Strains, Growth Condition and Genome Sequencing and Assembly

The strain of *A. mycotoxinivorans* CICC 1454 was purchased from China Center of Industrial Collection (CICC), Building 6, No. 24 Courtyard, Jiuxianqiao Middle Road, Chaoyang District, Beijing, 100015). The fungus was cultured in Malt Agar (Malt extract 30 g/L, Agar 15 g/L), 37°C, pH 6 for 24 h. Considering that our strain was not exactly the same with Vekiru’s, we first performed a LC-MS/MS analysis of the degradation products of ZEA by the strain and verified the reported singal *m/z* 351 (Supplementary Figure 1). Mycelia were collected and sent to Biomarker Technologies Corporation (Shunjie Building, No. 12 Fuqian Street, Shunyi District, Beijing, China) for genomic DNA extraction, sequencing, and assembly. The experimental process was implemented according to the standard protocol provided by Oxford Nanopore Technologies (ONT) company, including sample quality control, library construction, library-quality detection, library sequencing, and other procedures (Jain et al., 2016). Particularly, genome sequencing was performed using the Nanopore and the Illumina platform. The error correction of subreads was conducted using Canu v1.5 (Koren et al., 2017) and assembled using wtdbg2 (Ruan and Li, 2020), and the second generation reads were aligned to the preliminary assembled genome for further error correction using Pilon (Walker et al., 2014).

### Annotation of Protein-Coding and Non-coding Components

Repeat sequences in the genome were first identified and masked using two modules, RepeatMasker (Tarailo-Graovac and Chen, 2009) and RepBase (Bao et al., 2015), integrated in MAKER (Cantarel et al., 2008; Holt and Yandell, 2011). The conserved proteins of *Basidiomycota*, who locates at the nearest neighborhood of *A. mycotoxinivorans* in the phylogenetic tree (see below) (Liu et al., 2015), from BUSCO (Seppey et al., 2019) dataset were then retrieved and used as templates to identify homologous protein-coding regions in the genome by MAKER. The results were then processed with the module maker2zff in MAKER and converted to a ZFF format output, which was used by the gene prediction program SNAP (Korf, 2004) to generate a series of species-specific hidden Markov models for protein-coding gene prediction for the genome. The protein sequences obtained were finally compared with NCBI non-redundant datasets (nr) by using BLASTP (Sayers et al., 2020), and the candidates with an alignment *E*-value greater than 1e-3 were screened out for further analysis. The non-coding genes were annotated with Rfam (Griffiths-Jones et al., 2003), giving predictions of both rRNAs and snoRNAs; the tRNA genes were identified using tRNAscan-SE (Chan and Lowe, 2019).

### Phylogenetic Analysis

Genomes of the strains in the same family as *Apiotrichum*, namely *Apiotrichum brassicae*, *Apiotrichum domesticum*, *Apiotrichum gamsii*, *Apiotrichum gracile*, *Apiotrichum laibachii*, *Apiotrichum montevideense*, *Apiotrichum porosum*, *Apiotrichum veenhuisii*, *Cutaneotrichosporon arboriformis*, *Cutaneotrichosporon curvatum*, *Cutaneotrichosporon cyanovorans*, *Cutaneotrichosporon daszewskae*, *Cutaneotrichosporon dermatis*, *Cutaneotrichosporon mucoides*, *Cutaneotrichosporon oleaginosum*, *Takashimella koratensis*, *Trichosporon akiyoshidainum*, *Trichosporon asahii*, *Trichosporon coremiiforme*, *Trichosporon faecale*, *Trichosporon inkin*, *Trichosporon ovoides*, are retrieved from NCBI.

The proteomic data of all but three species were predicted by the above-mentioned protein-coding component annotation method. The exceptional three species are *A.porosum, T. asahii*, and *C. oleaginosum*, whose proteome data can be downloaded directly from NCBI website. Orthologous proteins within different species were detected and clustered using the module proteinortho5.pl of the program Proteinortho (Lechner et al., 2011). Single copy of orthologous proteins are extracted and linked to one amino acid sequence using a Python3 script. Sequences are aligned using muscle (Edgar, 2004), then the phylogenetic tree is constructed using Minimum Evolution (ME) method and tested with bootstrap method with 999 replications. The plot of the phylogenetic tree was prepared using iTOL (Letunic and Bork, 2019).

### The Prediction of Detoxification Enzymes

Screening the genome for enzymes that may catalyze the desired type of mycotoxin degradation involves several steps: assigning the catalytic-type for each predicted protein, modeling the homologous structures for selected candidate enzymes, docking the enzymes with the required substrate mycotoxin, and finally output a scoring function for each candidate enzyme according to the docking evaluation results.

The assignment of catalytic-type was implemented by scanning the annotated protein sequences against a preprocessed enzyme sequence database using BLASTP with an *E*-value cut-off 1e-5. The enzyme sequences were first download from BRENDA database (Scheer et al., 2011), then they were clustered using CD-HIT (Fu et al., 2012) to eliminate redundancy, and the representative enzyme with specific EC number was selected from each cluster. The percentage of cluster truncation was identified as 90%, and the maximum length difference of class members was set as 60 amino acids. Proteins with specific EC numbers of interest were selected for further analysis, for example, sequences labeled with EC 3.1.1 were identified as carboxylesterase candidates. At this stage, specific catalytic motifs, if any, can be used for further screening. Only protein sequences identified by PfamScan (Bateman et al., 2004) that contain both [A/G]GxWxxxx[F/Y]P[G/M]xxxD (Fraaije et al., 2002) and FxGxxxHxxxW[P/D] (Riebel et al., 2013) are considered BVMOs and were retained for further structural modeling and docking analysis. When their homology with protein sequences in the Protein Data Bank (PDB) exceeded 30%, the 3D structures of the enzymes were then built using MODELLER (Webb and Sali, 2017). Giving the structure, the ligand-binding sites were predicted on these structures using P2RANK (Krivák and Hoksza, 2018) with default parameters. Then, AutoDock vina (Trott and Olson, 2010) was used to dock the desired substrate, ZEA and ZOM in this work, to the predicted ligand-binding sites. A scoring function was assigned to each enzyme as the calculated binding energy. Other constraints, such as the distance between the active center and the substrate, were also be used as an auxiliary scoring function to help enzyme candidate screening. This workflow process, called Genome-scaled Prediction of Substrate-specific Enzymes (GPSE), has been integrated into a pipeline with a Python3 script, which is available at https://github.com/JinyuanSun/GPSE.

### Specialized Bioinformatics Analysis

The predicted protein sequences of *A. mycotoxinivorans* were upload to EggNOG server 4.5 (Huerta-Cepas et al., 2016) for clusters of orthologous groups (COG) assignment for further protein function characterization. The CAZymes were predicted by scanning against the dbCAN database (Yin et al., 2012; Huang et al., 2018) using HMMER v3.3 (Eddy, 1998) with default parameters. PHI-related genes, CYP450s, and peptidases were predicted by scanning against PHI-base (Winnenburg et al., 2006, 2007; Urban et al., 2015), fungal cytochrome p450 database (Park et al., 2008) and MEROPS (Rawlings and Barrett, 1999; Rawlings et al., 2010, 2017), respectively, using BLASTP (Altschul et al., 1997) with an *E*-value cut-off of 1e-10. Transmembrane domains, signal peptides were prepared using TMHMM (Krogh et al., 2001) and TargetP-2.0 (Emanuelsson et al., 2000, 2007) with their default parameters. Biosynthetic gene clusters (BGCs) in the genome and the corresponding secondary metabolites were predicted using the webserver of the antiSMASH 5.0 fungal version (Blin et al., 2017, 2019). All parameters were default.

## Results

### Genome Sequencing and Assembly

After two rounds of error correction, 7,236 Mb raw sequence reads of *A. mycotoxinivorans* obtained from Nanopore sequencing platform gave the final genome sequence. The basic statistics of the sequencing is given in Table 1. The genome is assembled into 7 contigs with a total length of 30,749,651 bp, which is similar to that of most *Trichosporonaceae* fungi whose sizes fall in the range between 20 and 40 M. The genome has been uploaded to NCBI under Bioproject accession PRJNA633776 and Genome Assembly accession GCA_013177335.1.

**TABLE 1:** The statistical results of the genome assembly of *A. mycotoxinivorans*.

**Number of contigs**	**7**
Total length (bp)	30,749,651
N50 (bp)	7,496,769
N90 (bp)	3,077,865
Max length (bp)	10,254,415
Min length (bp)	137, 936
GC content (%)	57.57

### Phylogenetic Analysis

**FIGURE 1:**
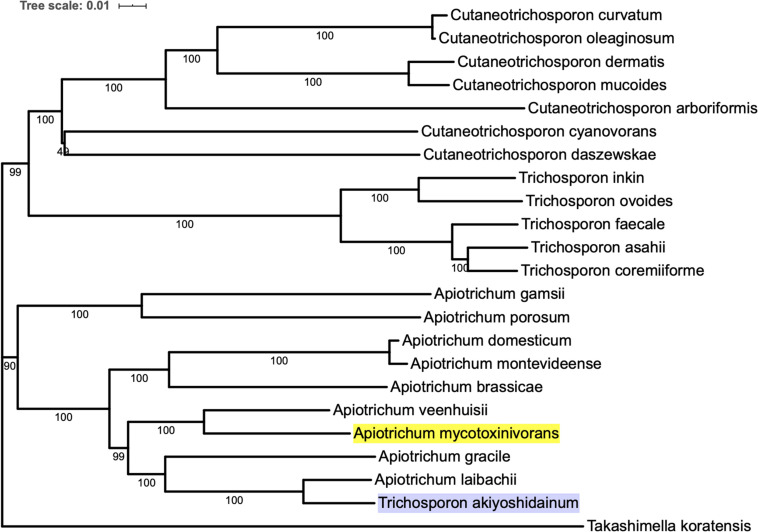
The phylogenetic tree was inferred based on orthologous amino acid sequences of the 23 selected Tremellomycetes genomes using Proteinortho v6, ME method is used in the phylogenetic tree, and the *T. koratensis* is the outgroup. The background color of *A. mycotoxinivorans* is highlighted with bright yellow and *T. akiyoshidainum* is highlighted with light purple.

In order to construct the phylogenetic tree of *A. mycotoxinivorans*, the genome sequences of 22 species of *Trichomonidae* (*Cutaneotrichosoron*, *Trichosporon* and *Apiotrichum*) were downloaded from the NCBI website, using *Takashimella koratensis* as the outgroup. For all species without the protein-coding sequences determined by experiments, we constructed SNAP hidden Markov model with Basidiomycota conservative proteins download from BUSCO and annotated the proteins from scratch. The phylogenetic tree was based on orthologous amino acid sequences of the 23 selected genomes and constructed with the program Proteinortho v6 with the *T. koratensis* as the outgroup. The results can be obtained on our website^1^. Phylogenetic tree analysis showed that *A. mycotoxinivorans* belongs to the genus *Apiotrichum* and is most closely related to *A. veenhuisii* (Figure 1). The profile of this phylogenetic tree is consistent with the phylogenetic classification of Tremellomycetes reported previously, and the classification is further confirmed (Liu et al., 2015). Also, in this phylogenetic tree, *T. akiyoshidainum* should be renamed as *A. akiyoshidainum.*

### Prediction and Annotation Coding and Non-coding Components

**FIGURE 2:**
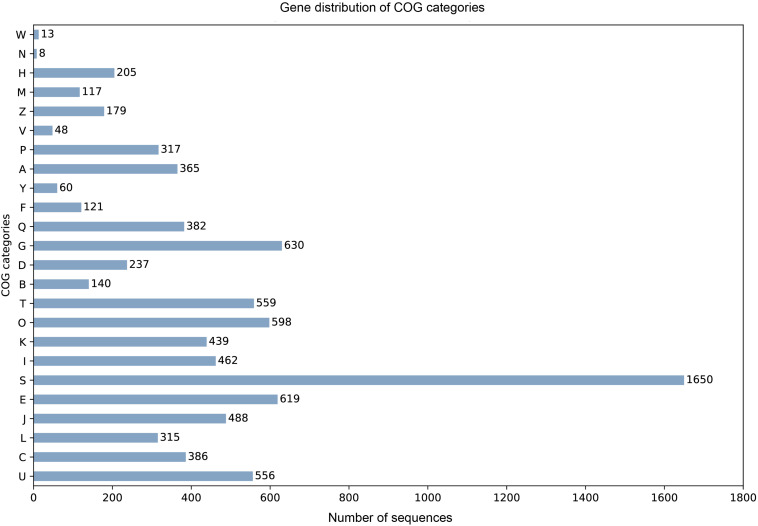
For the COG one-letter codes are Amino acid transport and metabolism [E]; Carbohydrate transport and metabolism [G]; Cell cycle control, cell division, chromosome partitioning [D]; Cell Motility [N]; Cell wall/membrane/envelope biogenesis [M]; Chromatin structure and dynamics [B]; Coenzyme transport and metabolism [H]; Cytoskeleton [Z]; Defense mechanisms [V]; Energy production and conversion [C]; Extracellular structures [W]; Function unknown [S]; General function prediction only [R]; Inorganic ion transport and metabolism [P]; Intracellular trafficking, secretion, and vesicular transport [U]; Lipid transport and metabolism [I]; Mobilome: prophages, transposons [X]; Nuclear structure [Y]; Nucleotide transport and metabolism [F]; Posttranslational modification, protein turnover, chaperones [O]; RNA processing and modification [A]; Replication, recombination, and repair [L]; Secondary metabolites biosynthesis, transport, and catabolism [Q]; Signal transduction mechanisms [T]; Transcription [K]; Translation, ribosomal structure, and biogenesis [J].

905 tRNA genes and pseudogenes were predicted by tRNAscan-SE 2.0 (Chan and Lowe, 2019), among them 835 tRNA genes contain 46 unique anti-codons. The number of 5.8S rRNA, 18S rRNA, and 28S rRNA were 1, 2, and 3, respectively. Due to the lack of transcription data, we combined the evidence from BUSCO database to train SNAP HMM model for protein-coding sequence prediction from scratch. Among the predicted 10922 protein sequences of *A. mycotoxinivorans*, 9404 homologous protein sequences were identified in NCBI NR database, 8669 of which could be assigned as orthologous seeds by eggnog-mapper v4.5.1. 8163 proteins were assigned a one-letter coded Clusters of Orthologous Groups of proteins (COGs) (Figure 2). 4693 sequences (54.13%) are assigned to gene ortholog (GO) terms. For detailed function prediction, more tools are needed. 1536 proteins predicted have transmembrane helix and 642 proteins with signal peptides.

### Carbohydrate-Active Enzymes Annotation

**FIGURE 3:**
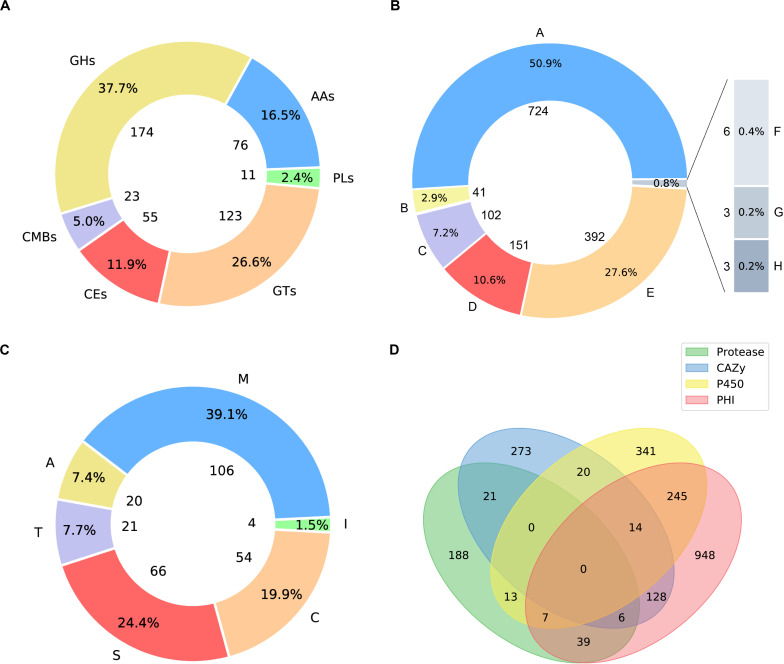
Pie plots of distribution of CAZymes, PHI related proteins and proteases and venn graph of intersection of proteses, CAZymes, PHI related proteins and CYP450. **(A)** 174 genes distributed in 45 families, which encode glycoside hydrolases (GHs) and 5 of them containing a carbohydrate-binding module (CBM). 123 glycosyltransferases (GTs) distributed across 27 families. 23 genes are encoding CBM proteins (CBMs), which belong to 7 families, and their main function is to bind to crystalized cellulose, granular starch, and other substrates. Also, there are 76 proteins with auxiliary activities (AAs), which involving in redox activities required by the breakdown of lignin, which implies *A. mycotoxinivorans* may be of redox activities. **(B)** Pie plot distribution of proteases. 106 genes distributed in 31 families, which encode metallopeptidases (M). 54 cysteine peptidases (C) distributed across 16 families. 66 genes are serine peptidases (S), which belong to 12 different families, 20 protein sequences annotated as aspartic peptidases (A), fall into 2 families. 21 proteins are predicted to be threonine peptidases (T) within 4 families. Also, there are 4 proteins are peptidase inhibitors, distributed in 25 families, which of great importance to maintain the balance within organisms. **(C)** Pie plot distribution of pathogen and host interaction (PHI). PHI classified as: reduced virulence (A), increased virulence or hypervirulence (B), lethal (C), loss of pathogenicity (D), unaffected pathogenicity (E), chemistry target: resistance to chemical (F), effector (plant avirulence determinant) (G), chemistry target: sensitivity to chemical (H). **(D)** Venn graph showing the intersections among the proteases (green), CYP450 enzymes (yellow), CAZymes (blue), and PHI proteins (pink).

Carbohydrate active enzymes (CAZymes) are essential enzymes to decompose the components of host cell walls during infection. Extracellular CAZymes are usually involved in plant adhesion and initial infection. The CAZy database records both catalytic and non-catalytic proteins involved in the synthesis and breakdown of complex carbohydrates and glycoconjugates. The database dbCAN-seq is a collection of CAZymes sequence and annotations (Huang et al., 2018). The analysis based on dbCAN-seq database shows that 462 genes encode CAZymes (Figure 3A), which are divided into 6 categories and 102 CAZy families. Among these genes, 59 proteins have transmembrane domains and 44 have signal peptides.

### Pathogenetic Proteins Involved in Pathogen-Host Interaction

*Apiotrichum mycotoxinivorans* is a human pathogen, especially in cystic fibrosis patients with lung infections (Almeida et al., 2017). To the best of our knowledge, although some relevant clinical cases have been reported, the pathogenic genes have not been successfully identified in *A. mycotoxinivorans* due to the lack of genomic resources. In order to obtain the putative genes associated with the pathogenicity and antibiotic resistance, we further analyzed the proteome of *A. mycotoxinivorans* using the pathogen-host interaction (PHI) gene database (Winnenburg et al., 2006, 2007; Urban et al., 2015). 1387 protein-coding genes were predicted to be associated with the pathogenicity of *A. mycotoxinivorans*, 53 of which are secretory proteins and 300 have transmembrane domains.

The distribution of PHI related proteins was predicted as follows (Figure 3B): 724 virulence decreased, 41 virulence increased or hypervirulent, 102 lethal, 151 pathogenicity lost, 392 pathogenicity unaffected, 3 chemistry targets: resistance to chemical, 3 effector (plant avirulence determinant), and 6 chemistry targets: sensitivity to chemical.

### Annotation of Proteases in Genome

Peptidases are enzymes that catalyze the hydrolysis of peptide bonds, which is very important for the survival of organisms. About 2% of the genes in the genome are encoded (Rawlings and Barrett, 1999; Rawlings et al., 2017). Peptidases are considered to destroy host tissues and provide nutrients for pathogen reproduction. Some extracellular peptidases are also involved in cell wall maintenance and remodeling, adhesion to the host’s external protective barrier, deregulation of the host protein hydrolase cascade, and inactivation of host antimicrobial peptides. The peptidase genes in *A. mycotoxinivorans* were annotated by using the MEROPS database (MEROPS 12.0, September 2017) (Rawlings et al., 2017). A total of 267 genes were predicted to be peptidases (Figure 3C), including metallopeptidase, cysteine peptidase, serine peptidase, aspartate peptidase and threonine peptidase, which is consistent with the distribution proportion of peptidase genes in the genome. In addition, four genes were predicted to be inhibitors. Among these genes, 11 proteins have transmembrane domains and 11 have signal peptides. These secreted peptidases might be involved in the infection of *A. mycotoxinivorans.*

### Cytochrome P450 Enzymes in *A. mycotoxinivorans*

Cytochrome P450 (CYP450) plays an important role in the primary metabolism of fungi, involving the integrity of cell wall and formation of the spore outer wall. So far, several CYP450s have been found in fungi, which catalyze reactions in the pathways of mycotoxin synthesis, hormone production, detoxification, fatty acid hydroxylation, etc. By scanning the CYP450 database of fungi (Park et al., 2008) with BLATP, we identified 640 putative CYP450s in *A. mycotoxinivorans*. Among them, 38 proteins were predicted to have signal peptides and 183 genes to have transmembrane domains. The cross-coverage of CYP450s, CAZymes, PHI proteins, and Peptidase is shown in Figure 3D.

### The Biosynthetic Potential of *A. mycotoxinivorans*

**FIGURE 4:**
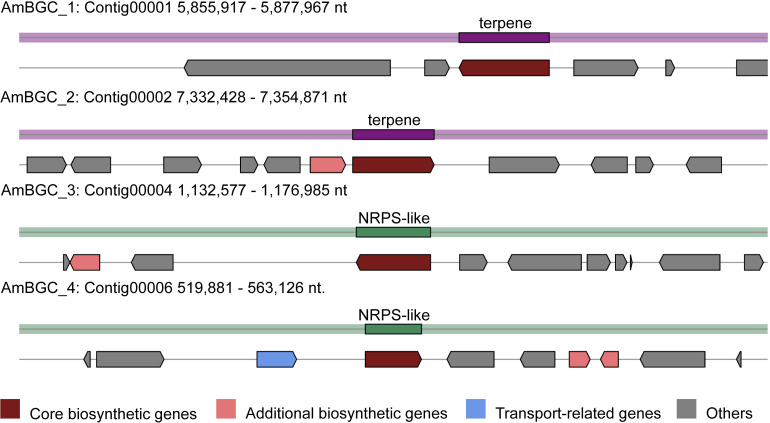
Predicted biosynthetic gene clusters (BGCs) in the *A. mycotoxinivorans*. Core biosynthetic genes are indicated in maroon, additional biosynthetic genes in light coral, transport-related genes in light steel blue, regulator genes in, and hypothetical protein-encoding genes in gray.

Many fungi can produce secondary metabolites, which can be drugs or precursors of drugs, with genes that are contiguously arranged in biosynthetic gene clusters (BGCs) (Rokas et al., 2020). We used the program antiSMASH 5.0 (Blin et al., 2019) to analyze the genome of *A. mycotoxinivorans* and identified 4 BGCs on the four different contigs (Table 2 and Figure 4). Although the amount of BGCs in mycoplasma is very small, AmBGC_1 was identified to produce a new terpenoid compound, because no similar BGCs have been found. The core gene of AmBGC_2 is 63.4% similar to that of oxidosqualene clavarinone cyclase (OCC) which is essential to clavaric acid biosynthesis in *Hypholoma sublateritium* (Godio et al., 2007). This suggests that *A. mycotoxinivorans* may be able to produce clavulanic acid, an anti-tumor product. No BGCs with significant similarity to AmBGC_3 and AmBGC_4 have been found. These BGCs may produce some new compounds, which need to be characterized by wet experiments in the laboratory.

**TABLE 2:** Four Biosynthetic gene clusters predicted in *A. mycotoxinivorans*.

**Cluster ID**	**Type**	**Gene number**	**Similar gene clusters number**
AmBGC_1	Terpene	6	0
AmBGC_2	terpene	11	1
AmBGC_3	NRPS-like	11	10
AmBGC_4	NRPS-like	10	1

### Prediction of Enzymes Involving Mycotoxin Degradation

**FIGURE 5:**
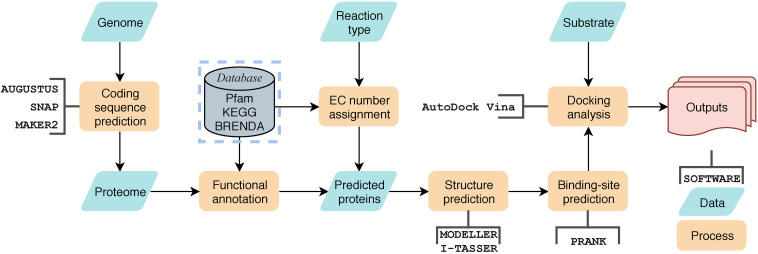
The general workflow of GPSE. Yellow blocks represent processes, green block represent data, open rectangle box represent software used in the process, the gray block represents database and the red block represents outputs.

As mentioned before, *A. mycotoxinivorans* can detoxify ZEA through the destruction of molecular structure rather than through masking. In order to screen mycotoxin degrading enzymes genome, we developed a workflow of Genome-scaled Prediction of Substrate-specific Enzymes termed as GPSE (Figure 5). Two BVMO candidate enzymes have been found in the genome using BVMO-specific sequence motifs (Mascotti et al., 2015), of which protein C1_1820 is the enzyme most likely to oxidize ZEA to the zearalenone oxidized metabolite (ZOM). The newly formed ester bond at C6’ in ZOM can be hydrolyzed by the carboxylesterase of *A. mycotoxinivorans*, which breaks the macrocyclic ring of ZEA at the ketone group at C6’. By comparing the predicted proteins with the enzymes in the Brenda database (Jeske et al., 2019), GPSE predicted 3157 hypothetical enzymes, of which 76 were carboxylesterases. We have built 42 predicted carboxylesterase homology models by the MODELER program (Webb and Sali, 2017) with a minimum sequence homology of 30%. ZOM was well docked to 32 binding sites in the 17 models, and it was found that the putative enzymes C2_44, C3_70, and C5_1043 were most likely to hydrolyze the newly formed ester bonds of ZOM by visual inspection.

## Discussion and Conclusion

*Apiotrichum mycotoxinivorans* is a very valuable fungus that can degrade and detoxify mycotoxin. It has been widely used in feed additives. However, it is also a human pathogen that can cause lung infection, especially in patients with cystic fibrosis. In this study, the whole genome of *A. mycotoxinivorans* was sequenced, assembled, and analyzed. The genome of *A. mycotoxinivorans* contains 30 million base pairs, which is similar to that of most of the sequenced Tremellomycete strains. In this work, we assembled the genome into 7 contigs, and considering that the seventh contig is very small, it should be part of other long chromosomes. We noted that researchers in Guangzhou Medical University had very recently uploaded genomic data of *A. mycotoxinivorans* GMU1709 with 6 contigs. Compared their genome data, the 6th and 7th contigs in this study are mostly likely to come from the same chromosome. We predicted the coding and non-coding components in the genome by interpreting the assembly data. The phylogenetic relationships of most of the sequenced strains in Tremellomycetes were analyzed by using the gene tree constructed from homologous proteins, which confirmed that the classification of *A*. *mycotoxinivorans* was consistent with the previous studies by Liu et al. (2015). The organization and distribution of tRNA genes and pseudogenes in genomes showed complex lineage-specific patterns.

In the genomes of all sequenced strains of *Apiotrichum*, *Cutaneotrichosporon*, and *Trichosporon*, the number of tRNA copies is very high, about 20∼40 copies per Mbp on average, which is much higher than that of *T. koratensis*. *T. koratensis* is used as an outgroup reference in phylogenetic analysis. The phylogenetic tree suggested that *T. akiyoshidainum* should be also classified as *A. akiyoshidainum.* Of 1387 pathogen-host interaction genes of *A. mycotoxinivorans*, 50% were categorized as virulence-related protein with reduced toxicity, followed by unaffected pathogenicity and pathogenicity loss, and 102 genes predicted to be lethal. Among 462 predicted carbohydrate enzymes, glycoside hydrolases (GHS) are the most common enzymes, followed by the glycosyltransferases (GTs) and auxiliary activities (AAs) which are redox enzymes acting together with CAZymes. *A. mycotoxinivorans* is not considered to be a plant pathogen, but the presence of AAs has the redox capability needed for lignin decomposition, indicating that it may have the potential to degrade agricultural wastes. The number of cytochrome-P450 enzymes and proteases was 640 and 267, respectively. Some proteases, which are also predicted to be PHI proteins, are likely to be involved in infection. *A. mycotoxinivorans* is sensitive to most antifungal drugs, which means these CYP450 variants are not resistant.

**FIGURE 6:**
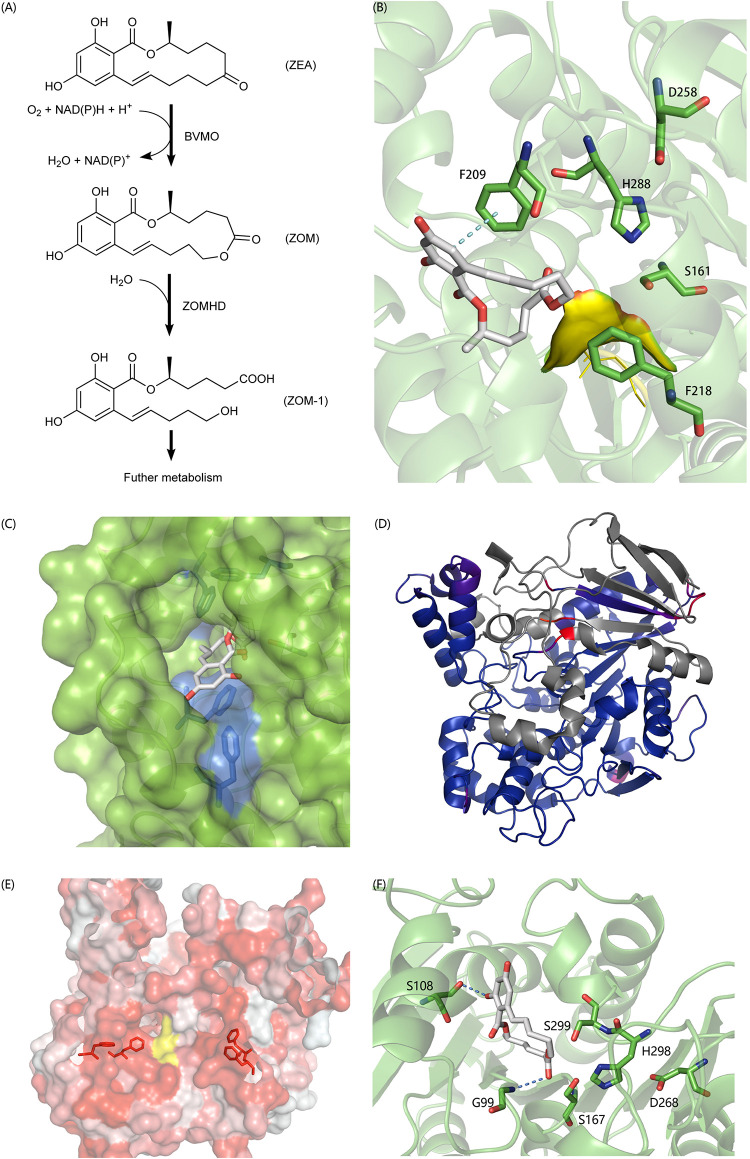
**(A)** The degradation process of ZEA. **(B)** Predicted binding pose of ZON to C2_44, the yellow surface is a highly flexible loop region G88-G89-G90 which may involve the formation of oxyanion hole. **(C)** The aromatic tunnel of C2_44, the surface is in green and the most hydrophilic surface is in blue. Aromatic amino acids within 4 Å of ZON were shown in stick. **(D)** The comparison of C3_70 and 4BE4, agreed parts in structures colored blue and extra parts colored in gray and identical but not agreed parts colored red and purple. **(E)** The aromatic flank of C3_70, the most hydrophobic surface is in red and the most hydrophilic surface is in white. The yellow surface is exposed part of catalytic triad. Aromatic amino acids on the surface forming the flank were shown in stick. **(F)** The binding poses of ZON to C5_1043. S108 and G99 involved formation of hydrogen bonds to stabilize the complex. S167-H298-D268 formed the typical catalytic triad. However, the S299 also in acceptable geometry forming a catalytic triad with H298-D268 and to carry out a nucleophilic attack on the carbonyl of the ester group. The structural figures were prepared using PYMOL (http://pymol.org).

In recent years, a lot of researches have been done on the biodegradation mechanism of ZEA, but most of them are based on the hydrolysis of the lactone bond of ZEA at C12’ (Peng et al., 2014; Qi et al., 2017) and the product ZOLs still has estrogenic biotoxicity. It was reported that *A. mycotoxinivorans* can degrade ZEA in two steps into a non-estrogenic compound (Vekiru et al., 2010; Figure 6A). First, it uses a BVMO enzyme to catalyze the insertion of an oxygen atom between C5’ and C6’, thus transferring the cyclic ketone to some zearalenone oxidized metabolite (ZOM). Next, some carboxylate ester hydrolase is recruited to hydrolyze ZOM, breaking the ester bond at C6’ to produce non-estrogen compounds (ZOM-1). However, as far as we know, the sequences of BVMO that oxidizes ZEA to ZOM (or ZEA-BVMO) and that of carboxylesterase that hydrolyzes ZOM (or ZOM-HD) have not been reported to be related to the oxidization and hydrolysis of ZEA at C6’. In this study, a workflow processing, called genome-scale prediction of substrate-specific enzymes (GPSE), was developed to screen ZEA-BVMO and ZOM-HD *in silico* from the genome of *A. mycotoxinivorans*. In particular, among the two predicted ZEA-BVMO candidates from GPSE, ZEA can dock in the substrate-binding pocket of C1_1820 structural model with an acceptable binding affinity of −5.9 kcal/mol, but it is difficult to dock with C2_51. Therefore, our results showed that C1_1820 is more likely to be the ZEA-BVMO enzyme. 32 putative ZOM-HDs predicted from GPSE were subjected to additional examination based on active-sites and protein-family annotation by using PfamScan. Three proteins were selected as putative ZOM-HDs as follows.

C2_44 belonging to the Abhydrolase_3 family (PF07859) has 49.8% homology with Est2349 from *Thalassospira sp.* GB04J01 (PDB ID: 4V2I). In the initial docking model, the aromatic ring in ZON was controlled by F209. Because the unfavorable position of the highly flexible loop G88-G89-G90 blocked the catalytic triad (S161-H288-D258), the distance between the carbon in the ester and S161 was 6.5 Å (Figure 6B). In addition, the tunnel surface is highly hydrophobic, which corresponds to another lactonase that hydrolyzes ZEA, and hydrophobic and aromatic interactions are important for substrate recognition (Figure 6C).

C3_70 is annotated as COesterase, a carboxylesterase belonging to protein family PF00135. It has 59.7% homology with a carboxylic esterase from *Ophiostoma piceae* [PDB ID:4BE4 (Gutierrez-Fernandez et al., 2014)]. However, despite the high homology between C3_70 and the esterase of *O. piceae*, C3_70 lacks a very long fragment, which is a cap domain in the hydrolase structure of *O. piceae* (Figure 6D). After carefully examining the *ab initio* gene predictions and ensuring that the missing part is not mistaken deleted for the intron, we think it is reasonable to assume that the function of cap deficient hydrolase is different from that of another enzyme. The lack of a cap may lead to a broader substrate spectrum. The surface around the active site is highly hydrophobic and forms aromatic side groups (Figure 6E), indicating that C3_70 can even hydrolyze ester bonds in polymers such as polyethylene terephthalate (PET) or lignin.

C5_1043 has 39.6% homology with HSL-like carboxylesterase of *Sulfolobus tokodaii*, which is annotated as the Abhydrolase_3 family. Among all the candidate hydrolytic enzymes identified by GPSE, C5_1043 has the highest binding affinity with ZEA, which was −9.5 kcal/mol. Because the surface of the substrate-binding pocket is not aromatic, the complex is stabilized by two hydrogen bonds and hydrophobic interactions (Figure 6F).

In summary, in the paper the genomic data of *A. mycotoxinivorans* were systematically and comprehensively analyzed to screen for ZEA-degrading oxidoreductases and hydrolases. We developed an annotation pipeline GPSE based on genomic data to predict substrate-specific enzymes, and established three-dimensional structure models to study the mechanism of oxidative degradation of ZEA catalyzed by monooxygenase. The workflow process GPSE developed in this study might help to accelerate the discovery of new enzymes for mycotoxin degradation.

## Data Availability Statement

The datasets presented in this study can be found in online repositories. The names of the repository/repositories and accession number(s) can be found in the article/Supplementary Material.

## Author Contributions

DM conceived and designed the research. JS and YX performed the experiments and contributed to the reagents, materials, and analysis tools. JS, YX, and DM analyzed the data. DM and JS wrote the manuscript. All authors read and approved the manuscript.

## Conflict of Interest

The authors declare that the research was conducted in the absence of any commercial or financial relationships that could be construed as a potential conflict of interest.

## Footnotes

1 https://minglab.tk/amzeadegrade.html

## Supplementary Material

FIGURE S1 Full-scan spectrum (ESI negative mode) of the ZEA metabolites. The 351 m/z is the signal of [M-H].
