# Supplementary figures and images for: Whole-Genome Sequencing and Bioinformatics Analysis of Apiotrichum mycotoxinivorans: Predicting Putative Zearalenone-Degradation Enzymes
Source: Front Microbiol. 2020 Aug 3;11:1866. doi: 10.3389/fmicb.2020.01866 (PMC7416605; doi:10.3389/fmicb.2020.01866)

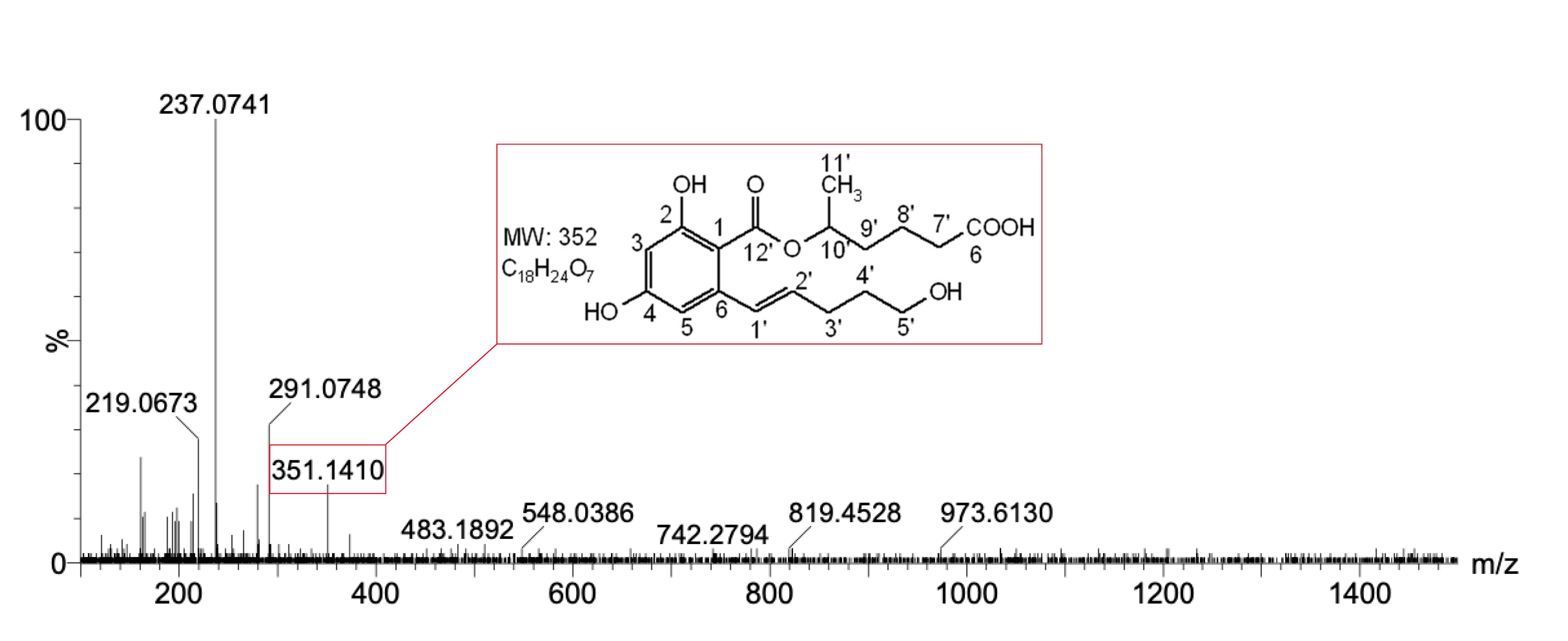

Supplement: FIGURE S1 — Full-scan spectrum (ESI negative mode) of the ZEA metabolites. The 351 m/z is the signal of [M-H]. [file Image_1.tif]
